# Medical News and Miscellany

**Published:** 1890-07

**Authors:** 


### Medical News and Miscellany

---

**Removed.**—Dr. E. S. Menefee has removed from Joshua to Granbarry.

---

**Small Pox** is raging at Saltillo, Mexico; and there has been a death at Laredo from the disease.

---

**Dr. R. M. Swearingen** and wife left for Europe via New York, on the first of July, on a three months' pleasure trip.

---

**Milk** from tuberculous cows is said to be killing infants in San Antonio, so says a telegram, on authority of Dr. McKillop, U. S. A.

---

**Dr. W. J. Westbrook**, of the firm of Drs. Roach & Westbrook, of Sipe Springs, was married in Galveston on June 26, inst., to Miss Hope Skeene of Mt. Pleasant.

Two deaths from sunstroke in Galveston on the 16th inst.

---

**Dr. W. M. Burger**, who went from Granger, Texas, to Montana, and had charge of the hospitals of the Tongue River Indian agency at Lame Deer, has returned to Texas and located at Claude, in Hopkins county.

---

The authorities of San Antonio have appointed a milch-cow inspector, or an inspector of cows and cows' milk, in view of the discovery, announced elsewhere, that children are being killed by the use of milk from tuberculous cows in that city.

---

**Heart Failure.**—The health-officer of Chicago has refused to recognize certificates of death from "heart failure." One hundred and fifty certificates so signed have been returned, with a request for more definite information as to the cause of death.—Exchange.

---

**Strophanthine as a Local Anæsthetic.**—It is claimed by M. Gley that strophanthine is superior to cocaine as a local anæsthetic. It relieves both tactile and thermic sensibility. Cocaine only relieves tactile sensibility.—Le Progre's Medicale, March 1, 1890.—Medical Bulletin, June.

---

**It Pays to Advertise in this Journal.**—"Our business in Texas has increased very materially recently, and we believe it is due in a large measure to our advertisement in your Journal."

PHENIQUE CHEMICAL CO.,
St. Louis, June 20.

[Voluntary and unsolicited.—ED.]

---

This issue begins Vol. 6 of DANIEL'S TEXAS MEDICAL JOURNAL. It has enjoyed five years of most remarkable success, and enters upon the sixth year with most flattering prospects. Thanking our friends for their generous support, the JOURNAL cordially invites a prompt renewal of subscriptions; and calls on all in arrears to please settle at once. . All original subscribers may know that their time is out; expired with the June number.

Three cases of malignant diphtheria—one death—occurred in a family six miles from Austin, in Dr. Morris' practice. No possible connection can be traced to any infected person or thing; and no local cause can be found. The water is from a clear, gushing mountain spring; what can be the cause?

---

Death of Dr. Towsey.—Dr. Samuel A. Towsey, one of the old "land marks" of Galveston, died in that city on the 11th inst. He was born in England in 1810; studied medicine and graduated in that country, and came to Texas in 1837, and entered the Texas navy, where he served several years. He had resided in Galveston upwards of fifty years.

---

Texas will be well represented at the Tenth International Congress at Berlin next month. Drs. Swearingen and Tyner from Austin (and their wives), Dr. Eastland from Wichita (and his wife), Drs. E. J. Beall, H. K. Leake and J. M. Pace, from Dallas, and Dr. H. Leonard, from New Braunfels, will be there as delegates from Texas State Medical Association.

---

Dr. T. J. Tyner, of Austin, left on the 15th inst. for Europe, via New York, on an extended tour. Mrs. Tyner accompanied him. At Berlin, in August, Dr. Tyner will read a paper before the International Congress, on an improved operation for cataract extraction, devised by himself. Dr. H Leonard, of New Braunfels, also left for Berlin, in company with Dr. and Mrs. Tyner.

---

For Chronic Vesical Catarrh.—

℞. Acidi nitrici pur, gutt iii.
Aqua dest. ℥vi.

M. S.—Inject in the bladder after first irrigating with warm water.

G. L. Bauer, M. D.,
St. Louis Clinique.

---

Messrs. Sharp & Dohme renew their advertisement for this, the sixth consecutive year, and request us to consider them permanent patrons. Messrs. Battle & Co. renew their ad. also, for the sixth consecutive year, and Dr. Bodine, Dean of the University of Louisville Medical College, renews also for the sixth round. Can anything better testify to the value of the JOURNAL as an advertising medium? Write for rates.

---

**Prince Bismarck.**—It is said that the Prince, when asked numerous questions as to his habits of life, etc., impatiently exclaimed to the young doctor who had been called to attend him: "Oh, be off, I don't like doctors who ask so many questions." The physician naturally took his leave, but, on reaching the door, gave the irascible potentate a word of advice, to the effect that he had better call in a veterinary surgeon, he being the only man who asked his patients no questions.—Med. Press and Record.

---

**Comparative Action of Iodides of Potassium and Sodium.**—Dr. Laborde (N. Y. Med. Record, Paris Cor. Lancet) calls attention to an error which is very prevalent in therapeutics, in considering isomeric substances as possessing the same physiological and therapeutical properties as their congeners. He says, the blood pressure is augmented by iodide of potassium, whereas iodide of sodium scarcely modifies it. Experiments upon lower animals showed that iodide of potassium produced general tetanus, whereas experiments with iodide of sodium, even with double doses, produced negative results. Iodide of potassium exites the nervous centers, whence its action on the vascular system and on the heart.

---

**Von Boeckmann,** "The" Bookmaker, requests us to announce that he will bind Volume V. (just completed) of DANIEL'S TEXAS MEDICAL JOURNAL, in beautiful and substantial style, for one dollar; that those having papers to appear in the Transactions desiring reprints, must communicate with him at once. The Transactions is now in press, and will be issued speedily—a handsome volume.

Von Boeckmann has printed this journal and the State Association Transactions five years, and given satisfaction. He has the most complete outfit south of St. Louis, and will duplicate St. Louis and Chicago prices on anything from a two-leaf folder to a 1000-page royal octavo book.

**Gone to Europe** — Dr. O. Eastland, ex-Vice-President of Texas State Medical Association, and his estimable wife, sailed for Europe June 18th, ult. The JOURNAL was favored with a letter from the doctor, written in mid-ocean and mailed at Queenstown. The doctor will attend the Tenth International Medical Congress, to which he is an accredited delegate.

The Association delegates who have gone, so far as reported to the JOURNAL, are: Drs. O. Eastland, R. M. Swearingen, H. Leonard, T. J. Tyner, E. J. Beall, B. E. Hadra, J. M. Pace and H. K. Leake. We wish them a happy time.

---

**The use of the Galvanic Current as a Laxative.**—John V. Shoemaker, A. M., M. D., of Philadelphia, in the Medical Bulletin for June, calls attention to the galvanic current as a laxative. An olive pointed electrode, negative pole, is introduced into the rectum, while the positive pole is placed on the perineum. The current is the strength of one milliampere. The desire to go to stool comes on within two minutes. It was ascertained that the use of the negative pole within the rectum caused relaxation of the sphincter muscle, while the positive pole produced contraction of this muscle. Chronic constipation may be robbed of its terrors by the use of the galvanic current. Its further use should be reported.

---

**A Cardiac Murmur of Itself of Little Significance.**—Dr. Beverly Robinson stated, at a recent meeting of the Practitioners' Society of New York, that the fact had come to be pretty generally recognized that a cardiac murmur of itself meant very little. It came down to the question whether the heart was doing its work well. There were certain ways of determining that point, aside frcm physical signs. One evidence of lack of proper compensatory power was difficult breathing; a second was palpitation; a third was pain in and around the heart. If no renal nor other physical evidence that the heart was suffering, the mere presence of a cardiac murmur should not be considered as of great importance.—Practice.

---

**Our new advertisements** are so numerous that it would be impossible to mention each one separately, and do justice to any in the small space at our disposal. We advise our readers to scan them closely. If they contained nothing to interest physicians, the parties would hardly pay to print them. It is a mistake to think the advertisements are not reading matter; they are, and are full of interest.

Especial attention is called to the large list of medical colleges advertised in this issue, as now is the time students are looking around to decide where they will attend the next course. In writing to any of the advertisers in the JOURNAL, we will take it as a favor if our readers will say where they saw the advertisement. Read all of of them!

---

The Treatment of Keloid.—A. G. Browning, M. D., of Maysville, Ky., (Med. Record, May 10, 1890), says Keloids are curable by the use of bichloride of mercury and collodion. He says, "I use the bichloride in the proportion of 1 to 30 of collodion. The tumor is thickly coated with this and allowed to remain until it exfoliates, four or five days generally. Another and another is appliied in this way, till by successive coatings the growth is reduced to a level with the surrounding surface, when the cure may be regarded as complete. From three to half a dozen applications, owing to extent of growth, have generally proven sufficient."

Dr. Browning says there is little or no irritation produced, and that the crust drops off, bringing with it a layer of the coagulated tissue which leaves the parts smooth and dry for the next coat. Warts and corns are cured by the same process.

